# Fabry Disease Beyond Storage: The Role of Inflammation in Disease Progression

**DOI:** 10.3390/ijms26157054

**Published:** 2025-07-22

**Authors:** Giuseppa Biddeci, Gaetano Spinelli, Paolo Colomba, Giovanni Duro, Irene Giacalone, Francesco Di Blasi

**Affiliations:** Institute for Biomedical Research and Innovation, National Research Council of Italy (IRIB-CNR), Via Ugo La Malfa 153, 90146 Palermo, Italy; giuseppa.biddeci@irib.cnr.it (G.B.); gaetano.spinelli@irib.cnr.it (G.S.); paolo.colomba@irib.cnr.it (P.C.); giovanni.duro@irib.cnr.it (G.D.); irene.giacalone@irib.cnr.it (I.G.)

**Keywords:** inflammation, Fabry disease, lysosomal storage disorder, NF-κB, p65 iso5

## Abstract

Fabry disease (FD) is a rare X-linked lysosomal storage disorder caused by mutations in the *GLA* gene, resulting in a deficient activity of the enzyme α-galactosidase A (α-Gal A). This deficiency leads to the progressive accumulation of globotriaosylceramide (Gb3) and its deacylated form, globotriaosylsphingosine (Lyso-Gb3), in various tissues, contributing to a broad spectrum of clinical manifestations. Recent evidence highlights the crucial role of inflammation in the pathophysiology of FD, influencing disease progression and clinical outcomes. This review provides a comprehensive overview of the relationship between inflammation and FD, with a particular focus on the impact of inflammatory processes on disease progression and complications.

## 1. Introduction

**Figure 1:**
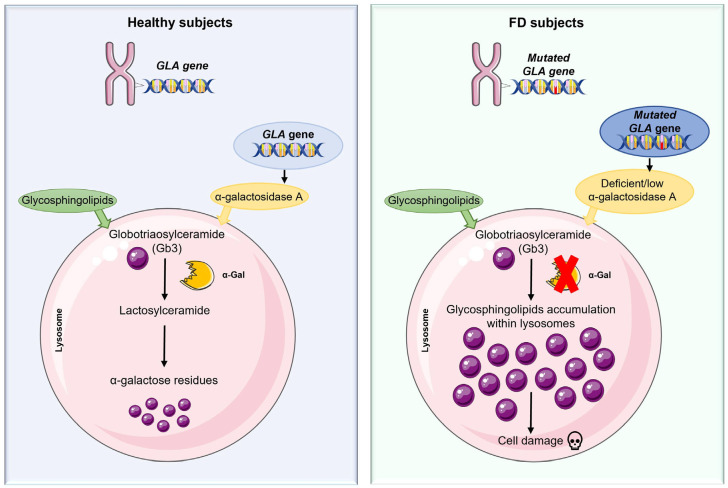
Deficiency of α-galactosidase A results in a progressive accumulation of the incompletely degraded Gb3 substrate in the cells, leading to cellular dysfunction.

Lysosomal storage diseases (LSDs) are a heterogeneous group of inherited metabolic disorders characterized by deficiencies or malfunctions of lysosomal enzymes, resulting in the accumulation of undegraded substrates within lysosomes and consequent cellular and tissue damage [1]. To date, more than 70 diseases have been identified as caused by deficiencies or malfunctions of lysosomal enzymes [2]. Among LSDs, Fabry disease (FD, Online Mendelian Inheritance in Man (OMIM) #301500) has attracted particular attention due to its wide-ranging clinical manifestations, therapeutic challenges, and the identification of inflammation as a key pathogenic component [3]. FD, also known as Anderson-Fabry disease, was first described in 1898 by Johannes Fabry and William Anderson. It is currently recognized as the second most common LSD after Gaucher disease [4] and is an X-linked inherited disorder caused by mutations in the galactosidase alpha (*GLA*) gene, located on the long arm of the X chromosome (Xq22.1) [5,6]. This gene encodes the lysosomal hydrolase α-galactosidase A (α-Gal A). Deficiency of α-Gal A leads to the progressive accumulation of globotriaosylceramide (Gb3) and its soluble derivative, globotriaosylsphingosine (Lyso-Gb3), in various cell types (Figure 1). The accumulation of these substrates within lysosomes affects multiple organs and tissues, including endothelial cells, renal glomeruli and tubules, cardiac myocytes, ganglion cells of the nervous system, smooth muscle cells, corneal epithelial cells, and blood vessels, resulting in a progressive multi-system disorder [7,8,9,10]. FD is primarily classified as a LSD due to substrate accumulation, with therapeutic strategies aimed at reducing lysosomal burden [11,12,13]. Clinical observations indicate that FD is characterized not only by lysosomal enzyme deficiencies. In fact, increasing evidence suggests that inflammatory mechanisms significantly contribute to disease progression and clinical manifestations. Substrate accumulation within lysosomes alters normal physiological functions, including the innate immune response [14,15], antigen presentation [16,17,18], release of inflammatory mediators, and phagocytosis [16,17,18,19], altered lipid trafficking, endoplasmic reticulum (ER) stress, autophagy, and oxidative stress [20,21,22,23].

The Gb3 and lyso-Gb3 accumulation has been shown to activate pro-inflammatory pathways, including Toll-like receptor 4 (TLR4) signaling and nuclear factor-kappa B (NF-κB) activation [24,25,26], resulting in the release of inflammatory cytokines such as tumor necrosis factor-alpha (TNF-α) [27], interleukin-6 (IL-6), and monocyte chemoattractant protein-1 (MCP-1) [28,29,30]. The central role of nuclear factor-kappa B (NF-κB) signaling in the inflammatory processes underlying FD has recently been highlighted. In particular, the role of the NF-κB isoform, p65 iso5, has been investigated in the inflammatory profile of FD, demonstrating a differential expression pattern in peripheral blood mononuclear cells (PBMCs) of affected individuals compared to healthy controls, supporting its potential involvement in the disease’s chronic inflammatory state [31,32]. Several studies have reported the involvement of both the innate and adaptive immune systems in FD patients, along with altered cytokine profiles [25,29,33,34,35,36], increased infiltration of immune cells in affected tissues, and the presence of chronic low-grade systemic inflammation, even in individuals receiving therapies [25,26]. All these processes negatively affect normal cellular functions, leading to systemic manifestations. Given the progressive nature of FD, early diagnosis is essential to reduce disease complications and improve quality of life. Although advances in therapeutic strategies, including enzyme replacement therapy (ERT) and chaperone therapy, have provided new opportunities for disease treatment, major challenges remain in identifying novel therapeutic targets to further improve patient outcomes [37]. The aim of this review is to comprehensively investigate the correlation between lysosomal dysfunction, glycosphingolipid accumulation, and inflammation in FD and to explore the mechanisms linking inflammation to disease progression. To provide a comprehensive and updated overview of the role of inflammation in FD, we conducted an in-depth analysis of the most relevant and recent scientific literature. Particular emphasis was placed on studies exploring inflammatory mechanisms and their contribution to disease progression and organ damage. Selected articles were critically evaluated to offer a pointing discussion on the interplay between lysosomal dysfunction, glycosphingolipid accumulation, and chronic inflammation.

## 2. Fabry Disease: Phenotypic Variability

**Figure 2:**
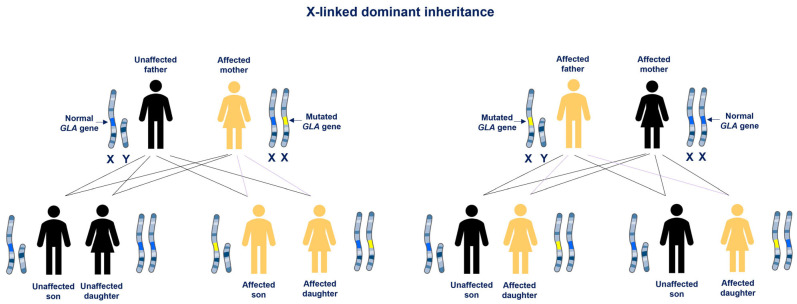
X-linked dominant inheritance. In FD, affected mothers have a 50% risk of passing the mutated *GLA* gene to their children regardless of gender, while affected fathers pass the defective *GLA* gene to all of their daughters and to none of their sons.

The clinical manifestations of FD progress gradually and show considerable variability in age of onset, severity, presentation, and disease course. These prognoses depend on several factors, including the degree of residual α-Gal A activity, patient gender, and genetic variability [10]. The constant accumulation of Gb3 and lyso-Gb3 is associated with a wide range of signs and symptoms, including cardiovascular dysfunction, dermatological manifestations, neuropathy, and kidney failure [38,39]. Two main phenotypes are recognized, the classic form, characterized by enzymatic activity less than 3% of normal, and the late-onset form, with residual but not null enzymatic activity (>3% and <30%) [6]. The classic form, also known as early-onset, is characterized by very low or absent α-Gal activity and multi-organ involvement, mainly in males. The late-onset or atypical forms often presents with milder symptoms and isolated organ involvement, such as cardiac or renal disease [38,40]. Patients with the classic form typically exhibit symptoms from early childhood or adolescence such as hypohidrosis, chronic neuropathic pain, lenticular and corneal opacities, cutaneous angiokeratomas, gastrointestinal symptoms, and microalbuminuria or proteinuria. With increasing age, these symptoms progress to cardiomyopathy, cerebrovascular involvement, renal failure, and early death [6,41,42,43]. In the late-onset form, symptoms appear between the third and sixth decades of life and patients may not exhibit the early symptoms typical of the classic form, complicating diagnosis. Due to its X-linked inheritance, affected males can only transmit the mutation to their daughters, while heterozygous females have a 50% chance of passing it to both their daughters and sons (Figure 2) [6].

Furthermore, males usually present more severe symptoms, while heterozygous females exhibit a highly variable phenotype due to the phenomenon of X-chromosome inactivation (XCI), also known as lyonization [44]. In hemizygous males, FD symptoms can present in childhood or adolescence, with systemic involvement progressing in stages [39]. Females may show a wide variety of signs and symptoms, ranging from mild to severe, with α-Gal A activity ranging from low to normal [45]. This variability is attributed to random inactivation of one X chromosome in each somatic cell during embryonic development [4,6,46]. Since heterozygous females have milder symptoms at a later age of onset than males, the distinction between classic and late-onset forms is mainly applicable to male patients, who tend to have a more severe phenotype due to their hemizygous status. The extent of XCI plays an important role in determining disease severity among female patients. The type of *GLA* gene mutation may also influence clinical presentation, with some mutations linked to early-onset and others, mainly missense mutations, associated with late-onset disease [6,43]. Considering phenotypic variability among individuals with the same mutation type, it is very challenging establishing a clear correlation between mutation and FD phenotype [47,48]. In addition to the classic and atypical variants, the presence of genetic variants of unknown significance (GVUS) should also be considered, as they further complicate the diagnosis and classification of FD. Although these variants cannot be definitively classified as pathogenic, such as the classic or late-onset forms, they may still be associated with clinical symptoms of FD. Patients carrying GVUS often present with normal α-Gal A enzymatic activity and normal lyso-Gb3 levels, yet may still exhibit manifestations consistent with FD, thereby adding complexity to the diagnostic process. The correlation between specific *GLA* mutations and the phenotypic expression among individuals with the same mutation can vary significantly, with consequent complexity of genotype-phenotype correlations in FD [49].

### Diagnosis

**Figure 3:**
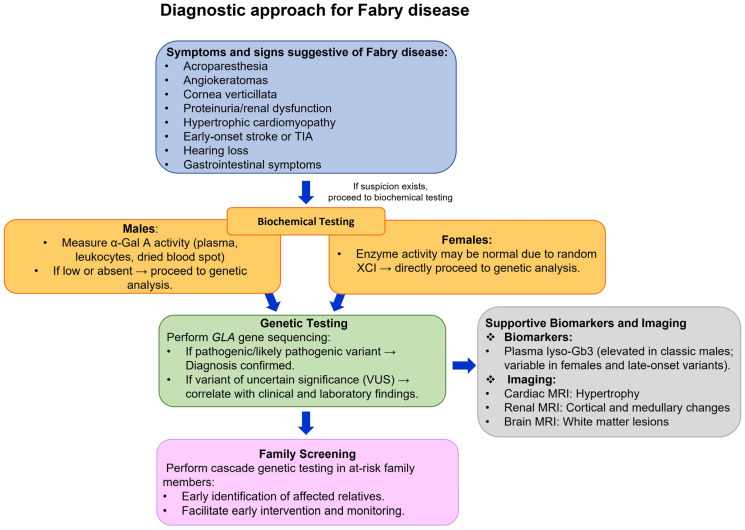
Schematic representation of the diagnostic approach for FD, highlighting clinical manifestations, biochemical testing, genetic analysis, the role of supportive biomarkers and imaging, and the importance of family screening. Abbreviations: α-Gal A, α-galactosidase A; lyso-Gb3, globotriaosylsphingosine; MRI, magnetic resonance imaging; TIA, transient ischemic attack; XCI, X-chromosome inactivation.

The diagnosis of FD requires a comprehensive and integrative approach, including biochemical, genetic, and clinical evaluations. Due to the heterogeneous clinical manifestations and similarities with other diseases, the diagnosis remains challenging [50]. In particular, diagnosis in females and individuals carrying genetic variants can be complicated by variable clinical expression and incomplete penetrance [51]. A thorough diagnostic approach is recommended, including detailed medical and family history, physical examination, clinical and biochemical assessments, genetic testing, and imaging procedures (Figure 3). Some clinical manifestations, such as acroparesthesia, angiokeratomas, and cornea verticillata, are highly specific for the disease [52,53,54]. In hemizygous males, diagnosis begins with quantitative assessment of α-Gal A activity in plasma, serum, dried blood spot (DBS), or leukocytes, typically revealing markedly reduced or absent enzyme levels [55,56,57,58]. The identification of a hemizygous pathogenic variant in the *GLA* gene by molecular genetic testing provides definitive confirmation in male probands. In affected females, α-Gal A activity can range from low to normal due to random XCI, making enzymatic assays alone insufficient for diagnosis. Therefore, genetic analysis is required for certain diagnosis in females [10,59,60]. The identification of a pathogenic *GLA* mutation is essential for definitive diagnosis in both sexes and is considered the gold standard for diagnosis and also facilitates screening of at-risk family members. Measurement of Lyso-Gb3 has emerged as a useful biomarker for classic FD, although its sensitivity is limited in heterozygous females and individuals with non-classical phenotypes [61,62,63,64]. Unfortunately, FD diagnosis is often delayed, frequently occurring when irreversible organ damage has already developed. Early diagnosis is important for facilitating genetic counseling and implementing preventive strategies in affected families. Given the progressive and multisystemic nature of the disease, early and accurate diagnosis based on a combination of biochemical, molecular, and clinical criteria, supported by expert interpretation, is essential to initiate rapid therapeutic interventions and prevent irreversible organ damage.

## 3. Inflammation in Lysosomal Storage Disease

Lysosomes are essential organelles within the cellular clearance system, but they also play important roles in several cellular processes including energy balance, mitochondrial metabolism, cellular lipid trafficking, regulated cell death, and inflammation [25,65,66]. Inflammation is the innate defense response to harmful stimuli such as pathogens, damaged cells, toxic compounds, or irradiation [67], aiming to remove injurious agents and initiate the healing process [68]. The inflammatory response can be defined as the coordinated activation of signaling pathways that regulate the levels of inflammatory mediators in both resident tissue cells and inflammatory cells recruited from the bloodstream [69,70,71]. While the primary pathology of LSDs arises from specific enzymatic deficiencies, increasing evidence suggests that inflammation plays a central role in the pathogenesis of many LSDs. The storage of undegraded macromolecules acts as a trigger for chronic immune activation, exacerbating tissue damage and significantly contributing to organ dysfunction [2,25,72].

### 3.1. Inflammation in Fabry Disease and Molecular Mechanisms

**Figure 4:**
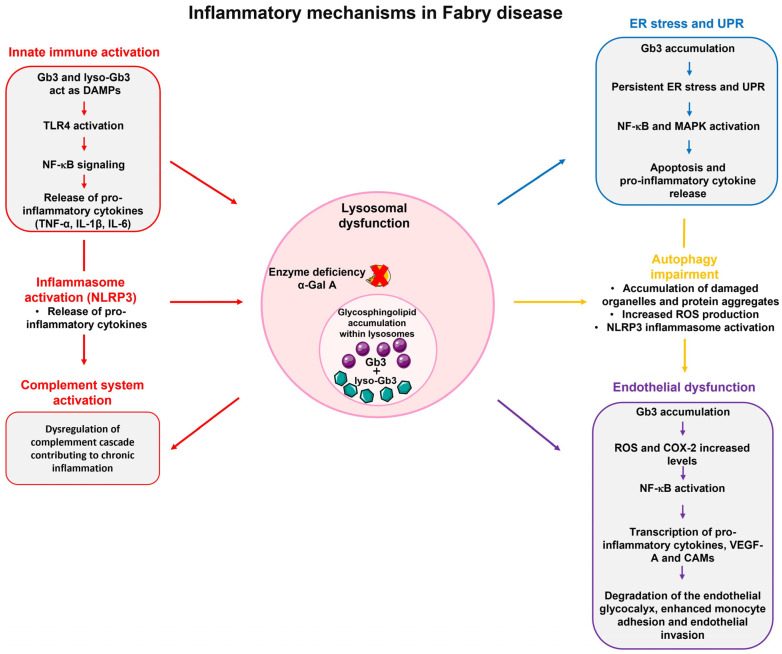
Inflammatory mechanisms involved in Fabry disease. Lysosomal dysfunction resulting from α-galactosidase A (α-Gal A) deficiency leads to the accumulation of glycosphingolipids, primarily globotriaosylceramide (Gb3) and its deacylated form globotriaosylsphingosine (lyso-Gb3). This accumulation triggers several inflammatory pathways. In the innate immune system activation, Gb3 and lyso-Gb3 activate the toll-like receptor 4 (TLR4) and nuclear factor-kappa B (NF-κB) signaling pathway, promoting the release of pro-inflammatory cytokines such as tumor necrosis factor-alpha (TNF-α), interleukin-1 beta (IL-1β), and interleukin-6 (IL-6). NOD-, LRR- and pyrin domain-containing protein 3 (NLRP3) inflammasome activation and complement system dysregulation further amplify the inflammatory response. Furthermore, Gb3 accumulation induces persistent endoplasmic reticulum (ER) stress and unfolded protein response (UPR), which contributes to cytokine release and apoptosis. Autophagy impairment leads to increased production reactive oxygen species (ROS) and further inflammasome activation. Additionally, endothelial dysfunction, characterized by NF-κB activation and upregulation of vascular endothelial growth factor A (VEGF-A) and cell adhesion molecules (CAMs), promotes vascular inflammation. These interconnected mechanisms promote chronic inflammation and contribute to organ damage in FD. Abbreviations: MAPK, mitogen-activated protein kinase; COX-2, cyclooxygenase-2.

In FD, lysosomal dysfunction has several downstream consequences that promote a pro-inflammatory microenvironment. The accumulation of undegraded substrates, such as Gb3 and lyso-Gb3, act as danger-associated molecular pattern (DAMP), activating pattern recognition receptors (PRRs), especially Toll-like receptors (TLRs), on immune and parenchymal cells [30,73]. Defects in autophagy lead to the accumulation of damaged mitochondria, which release mitochondrial DNA (mtDNA) and reactive oxygen species (ROS) that activate inflammasomes and NF-κB signaling [74]. Lysosomal stress can also cause the leakage of cathepsins and other proteases into the cytosol, triggering caspase activation and cell death pathways, including pyroptosis [75]. Furthermore, lysosomal stress enhances the secretion of pro-inflammatory extracellular vesicles which promote inflammation and fibrosis in distant tissues [76]. Given that sphingolipid deposits alone do not completely explain the pathophysiology and intra-familial phenotypic variability of FD, it has been hypothesized that Gb3 buildup could trigger cellular mechanisms responsible for disease manifestations. Due to the persistent accumulation of unmetabolized glycolipid substrates, which continuously trigger inflammatory responses, FD is also characterized as an autoinflammatory disorder [25]. Inflammation in FD is believed to arise from multiple interconnected processes, including lysosomal dysfunction, oxidative stress, endothelial damage, and immune system activation. These mechanisms underlie several systemic manifestations of FD, such as neuropathic pain, cardiac dysfunction, and progressive nephropathy, suggesting a potential therapeutic target (Figure 4).

### 3.2. Immune System Activation in Fabry Disease

A very important role in FD pathogenesis is carried out by the inflammatory cascade triggered by damaged cells and dysregulated cellular functions, mediated by both the innate and adaptive immune systems. It has been demonstrated that Gb3 and lyso-Gb3 accumulation activate TLRs and inflammasomes, leading to stimulation of the innate immune response for immediate non-specific defense [36,77]. TLRs are key components of the innate immune system and are predominantly expressed by dendritic cells, macrophages, monocytes, neutrophils, and epithelial cells. These receptors are activated upon recognizing pathogen-associated molecular patterns (PAMPs) as well as DAMPs, which are endogenous signals released during cellular stress or injury [78,79]. In FD, undegraded substrates act as DAMPs, triggering innate immune system activation and contributing to the inflammatory response [80]. Specifically, lyso-Gb3 has been shown to activate TLR4, which subsequently triggers the NF-κB signaling pathway, leading to the release of pro-inflammatory cytokines, such as TNF-α, IL-1β, and IL-6. This TLR4-mediated mechanism underlies the contribution of innate immune activation to the chronic inflammatory state observed in FD [81]. Studies have shown elevated levels of pro-inflammatory cytokines, IL-6 and IL-1β, in patients with FD compared to healthy controls. Notably, this inflammatory response can be effectively blocked by using a TLR4-specific antibody, highlighting the crucial role of TLR4 in this process [35]. In addition to increased levels of pro-inflammatory cytokines such as IL-6, TNF-α, and IL-17, higher expression levels of some anti-inflammatory cytokines such as IL-10 and TGF-β1 have also been observed, particularly in patients with missense mutations following ERT [82]. The complement system, an essential component of immune defense, is also strongly activated in FD. Its dysregulation triggers a chain reaction of inflammatory responses that worsens disease progression [82,83]. Inflammatory cells such as macrophages, mast cells, and neutrophils are recruited in response to cellular stress and pro-inflammatory cytokines. Histological analyses of heart and kidney tissues from Fabry patients have revealed the presence of macrophage-associated markers, including CD68, CD163, and CD45, indicating localized immune activation [25,84,85]. It should also be considered that, sex-related differences in immune responses may significantly influence both the pathophysiology and clinical variability observed in FD [86]. This difference in immune response is influenced by various factors, including sex hormones, genetics, and environmental influences. Females, who exhibit more robust innate and adaptive immune responses compared to males, often present a strong immune response that could be deleterious, causing autoimmune or inflammatory diseases—partly due to random XCI and sex-specific immune modulation [87,88]. These immunological differences, in relation to FD may impact the degree of residual inflammation and fibrosis, as well as the therapeutic response to ERT. Furthermore, sex differences in pharmacokinetics and pharmacodynamics may influence the efficacy and immunogenicity of therapeutic agents, including ERT [89,90]. For instance, differences in antibody formation against recombinant α-gal A, as well as in complement activation and cytokine profiles, could contribute to variable clinical responses [91]. Therefore, considering sex as a biological variable is essential not only for understanding disease mechanisms but also for optimizing individualized treatment strategies and improving clinical outcomes in both male and female FD patients.

### 3.3. Endoplasmic Reticulum Stress and the Unfolded Protein Response

In addition to activation of the innate immune system, other mechanisms by which Gb3 and lyso-Gb3 accumulation trigger inflammation have been identified. Gb3 accumulation is responsible for ER stress and the unfolded protein response (UPR), leading to the upregulation of molecular chaperones, attenuation of protein translation, and increased degradation of misfolded proteins through ER-associated degradation (ERAD) [92]. Persistent ER stress and UPR failure results in apoptosis and activation of inflammatory pathway such as NF-κB and mitogen-activated protein kinase (MAPK), increasing pro-inflammatory cytokine production [93]. Studies have demonstrated that some mutations in α-Gal A induce significant ER stress, with the mutated enzyme predominantly retained in the ER, triggering a robust UPR [94,95]. This ER stress, rather than increased proteasomal degradation, impairs the α-Gal trafficking to lysosomes, suggesting that a negative gain-of-function underlies the pathogenesis for certain forms of FD [95]. The effect of lyso-Gb3 on neuronal cells (SH-SY5Y) was evaluated, and the results suggested that lyso-Gb3 treatment causes ER stress with negative effect on protein folding and proteasomal targeting by ubiquitination [96]. Many FD pathogenic variants are missense, and mutated proteins are misfolded and retained in the ER instead of being transported to the lysosome. Consolato et al. demonstrated that α-Gal A missense mutations resulted in protein misfolding and subsequent retention within the ER, with consequent ER stress and activation of the UPR, as evidenced by increased levels of key ER chaperones and stress markers [94]. It has become more evident that UPR signaling is essential in immunity and inflammation and that there is a mutual regulation between ER stress and inflammation. The ER stress can activate inflammatory pathways, and, on the other hand, pro-inflammatory stimuli can trigger ER stress, such that the resulting UPR activation can further amplify inflammatory responses [36,97,98]. These results indicate that FD is not only a LSD, but it is also correlated with ER stress-related mechanisms due to UPR, suggesting a new pathogenic pathway [94].

### 3.4. Impairment of Autophagic Processes

Several data suggest that Gb3 accumulation also disrupts autophagic processes, resulting in the accumulation of damaged organelles and protein aggregates. This impairment, as observed in cultured vascular endothelial cells, resulted in increased intracellular ROS production in a dose-dependent manner [99]. It also contributes to activation of the NOD-, LRR- and pyrin domain-containing protein 3 (NLRP3) inflammasome, linking autophagy dysfunction to inflammatory responses and pyroptotic cell death [24]. Impaired autophagy is also associated with mitochondrial dysfunction, recognized as an important factor in the pathogenesis and inflammatory profile of FD. Gb3 and lyso-Gb3 interfere with lysosomal function and impair mitochondrial homeostasis, leading to reduced membrane potential, altered mitochondrial morphology, and impaired oxidative phosphorylation and ATP production [100,101]. These defects increase ROS production, which promotes inflammation through the activation of redox-sensitive signaling pathways. Mitochondrial damage can also induce the release of mtDNA and ROS into the cytosol, triggering innate immune responses via NLRP3 inflammasome activation and stimulating the secretion of pro-inflammatory cytokines such as IL-1β and IL-18 [102,103,104]. Studies on human renal tubular epithelial cell lines isolated from FD patients, showed that impaired mitochondrial function results in severe disturbance of mitochondrial energy metabolism [105]. An in vitro study demonstrated that lyso-Gb3 induces ROS production through a receptor-interacting protein kinase 3 (RIPK3)-dependent mechanism, which subsequently impairs mitochondrial function by inhibiting the activity of respiratory chain complexes I and III [80].

### 3.5. Endothelial Dysfunction

Endothelial dysfunction is a well described aspect of FD. Gb3 increase the expression of cyclooxygenase-2 (COX-2), a pro-inflammatory enzyme that contributes to endothelial inflammation. This enzyme is also associated with elevated ROS levels, forming a feedback loop that further impairs endothelial function [99]. Elevated levels of the pro-angiogenic markers in Fabry patients’ serum support the hypothesis that Gb3 accumulation directly contributes to vascular damage. Exposure of vascular endothelial cells to Gb3 leads to increased ROS production and higher expression of adhesion molecules, facilitating the recruiting inflammatory cells [106]. In Fabry patients, the expression of the pro-angiogenic vascular endothelial growth factor A (VEGF-A), produced by endothelial cells, is significantly higher than in controls. This increased expression correlates with specific *GLA* mutations causing severe organ involvement [107]. Gb3 and lyso-Gb3 deposits in the vascular wall are thought to promote smooth muscle cells proliferation, causing arterial wall remodeling and lumen narrowing [108]. Studies comparing the effects of Gb3 accumulation to *GLA* deficiency in human cardiac endothelial cells have shown that Gb3 loading impairs several critical endothelial pathways, while *GLA* gene silencing alone does not appear to impact these pathways [109]. This suggests that endothelial dysfunction in FD is primarily due to Gb3 accumulation rather than the absence of *GLA* activity. In particular, this affects microvascular endothelial cells, altering their phenotype toward a vasoconstrictive and pro-inflammatory state [110]. The transcription of cell adhesion molecules (CAMs), induced by ROS and mediated by NF-κB contributes to the FD vasculopathy [111]. E-selectin, intercellular adhesion molecule 1 (ICAM-1), and vascular cell adhesion molecule 1 (VCAM-1) promote leukocyte rolling and adhesion at the endothelial level, initiating arterial wall infiltration and damage [106]. Oxidative stress activates the NF-κB signaling pathway, which plays an important role in inflammation and fibrosis via the induction of pro-inflammatory cytokines including IL-6, TNF-α, and IFN-γ [81]. Lyso-Gb3 and pro-inflammatory cytokines further contribute to endothelial dysfunction by activating NF-κB signaling, promoting monocyte adhesion, glycocalyx degradation, and vascular inflammation [112]. A proposed mechanism suggests that lyso-Gb3 and pro-inflammatory cytokines activate NF-κB signaling, triggering chronic inflammation, upregulating heparanases and monocyte-binding receptors. The subsequent increase in monocyte adhesion and endothelial invasion contributes to the development of endothelial dysfunction in FD [112]. The progression of these chronic disorders leads to irreversible tissue injury and eventually fibrosis. Despite growing knowledge, the precise connection between the underlying metabolic defect and tissue injury remains poorly understood. It is thought that the initial metabolic imbalance promotes the production of secondary mediators of injury that lead to inflammation, parenchymal cell loss, and fibrosis [41]. These findings highlight the complex pathophysiology of FD, where inflammatory response is driven by the accumulation of glycolipids, with significant contributions from immune system activation, ER stress, autophagy and endothelial dysfunction. The connection between these mechanisms intensifies the chronic inflammatory state, leading to systemic manifestations and progressive organ damage. Understanding these molecular processes could be important for the identification of novel therapeutic targets and to improve the treatments for FD and evaluate new strategies addressing not only the metabolic defect but also the underlying inflammatory response to reduce the disease progression and improve patient outcomes.

## 4. Clinical Implications of Chronic Inflammation in Fabry Disease

**Figure 5:**
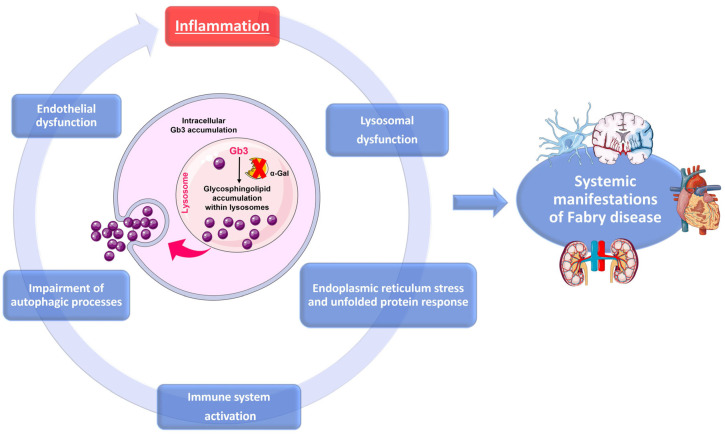
Inflammatory cascade and cellular dysfunction in FD. Progressive accumulation of glycosphingolipids, mainly Gb3, due to deficient α-Gal A activity, results in lysosomal dysfunction and secondary cellular stress responses, including ER stress and impairment of autophagy. These changes activate the immune system and cause endothelial dysfunction, creating a self-perpetrating inflammatory loop. This cycle exacerbates the systemic manifestations of FD, affecting multiple organs such as the CNS, heart, and kidneys.

Among the various pathogenic mechanisms implicated in FD, chronic inflammation has emerged as a critical contributor to the progression of organ damage. Inflammatory processes may play a pivotal role in mediating the transition from metabolic dysfunction to irreversible tissue injury and failure [24]. Chronic inflammation is responsible for increased parenchymal cell damage and fibrosis, ultimately compromising the structural and functional integrity of organs such as the central nervous system (CNS), heart, and kidneys (Figure 5). Thus, inflammation is not just a secondary response but may represent a central mechanism driving the pathophysiological progression of FD [3]. Recent evidence further highlighted that, despite the reduction of glycosphingolipid accumulation achieved through ERT, many patients maintain a persistent pro-inflammatory profile, characterized by elevated circulating cytokines and complement system activation markers [82]. This condition may reflect individual differences in genetic background, residual enzyme activity, and disease progression at the start of therapy. The observed differences between treated and untreated patients highlight the need for a more comprehensive therapeutic approach. This approach should not only focus on substrate reduction but also aim to modulate inflammatory pathways and chronic inflammation, with the purpose of preventing organ damage and fibrosis in FD [33,82].

### 4.1. Chronic Inflammation and Fibrosis in Fabry Nephropathy

In FD, kidney involvement is a common and well-recognized complication. Fabry nephropathy (FN) is attributed to tissue damage induced by the accumulation of Gb3 and lyso-Gb3 within kidney cells, including podocytes, glomerular and tubular epithelial cells [113]. The severity of FN can vary significantly among individuals. However, it often follows a gradual course that may ultimately lead to chronic kidney disease (CKD) and, in many untreated cases, end-stage renal disease (ESRD) by the fifth decade of life [114]. Generally, the kidney involvement, particularly in untreated males, tends to follow three clinical phases [115]. The first phase usually begins in childhood or adolescence. It is characterized by glomerular hyperfiltration, which often goes unnoticed due to the absence of symptoms. The prevalence of glomerular hyperfiltration has been reported to be particularly high (50%) in younger patients with FD (≤40 years) [116,117]. Parapelvic cysts have been reported in 30% of patients and may also appear in the early stages, serving as another early feature of kidney involvement in FD [118,119]. Tøndel et al. reported that podocyte foot process effacement and intracellular Gb3 inclusions can begin very early in life [120]. As injury progresses, podocytes begin to detach from the glomerular basement membrane and are lost in the urine. This phenomenon, known as podocyturia, further contributes to glomerular dysfunction [121,122]. During the second stage, signs of kidney damage become more evident. Patients may develop proteinuria, lipiduria, and the presence of Maltese cross (MC) crystals in the urine, along with a reduced capacity to concentrate or dilute urine, indicating impaired renal tubular function [123]. The third phase is characterized by severe kidney damage, often coupled with complications affecting the vascular, cardiac and cerebral systems. In hemizygous males, ESRD is a frequent outcome during their third to fifth decades of life. Gb3 also accumulates in the renal collecting ducts, interfering with normal electrolyte and acid-base regulation and potentially leading to chronic metabolic acidosis, impaired potassium handling, and nephrogenic diabetes insipidus [124,125]. Beyond these structural and functional changes, FN is increasingly understood as a condition deeply connected to chronic inflammation. Accumulation of Gb3 appears to initiate a cascade of inflammatory responses, attracting immune cells such as neutrophils, macrophages, and dendritic cells into kidney tissue. This persistent, low-grade inflammation triggers the body’s response. However, instead of resolving the injury, it leads to ongoing tissue remodeling and fibrosis [126]. This fibrotic process involves the activation and migration of myofibroblasts, along with the deposition of extracellular matrix (ECM) components [127]. Both injured renal epithelial cells and infiltrating immune cells contribute to this process by stimulating the release of profibrotic mediators such as transforming growth factor-beta (TGF-β) and platelet-derived growth factor (PDGF). Myofibroblasts play a central role in fibrosis. They are found throughout the kidney interstitium, arterioles, and mesangial areas, and are believed to originate mainly from pericytes and resident fibroblasts [128]. Rozenfeld et al. showed that in kidney biopsies from patients with FD, proximal tubular epithelial cells are an important source of TGF-β, which promotes the activation of myofibroblasts within the glomeruli and vessels, driving progressive fibrosis [129]. TGF-β levels tend to rise in the presence of chronic inflammation, helping to explain its role in FN. Although in vitro studies suggest that podocytes can respond to TGF-β by producing ECM components, its expression has not been consistently observed in glomeruli from Fabry patient biopsies [130], implying that podocytes might not be the primary contributors to TGF-β-mediated fibrosis in vivo. Recent findings also indicate that Gb3 can directly activate immune cells such as dendritic cells and monocytes through TLR4, thereby reinforcing the chronic inflammatory state [35]. In experimental models, exposure of renal tubular cells to Gb3 has been shown to induce epithelial-to-mesenchymal transition (EMT) via TGF-β signaling. This leads to cell cycle arrest, senescence, apoptosis, and ultimately interstitial fibrosis [131]. Gb3 also activates the Notch1 pathway, which in turn triggers NF-κB signaling and chemokine production [81]. Moreover, Notch1 plays a role in EMT-related transcriptional programs that further enhance fibrosis in FN [84,132]. Together, these findings reveal that kidney fibrosis in FD is not just a consequence of storage material buildup, but the result of persistent and unresolved inflammation that drives tissue damage over time. The continuous presence of Gb3 and lyso-Gb3 activates immune responses, leading to the release of signals that gradually alter the kidney structure. This chronic inflammatory state accelerates loss of function. Recognizing inflammation as a central player in FN highlights the importance of treatments that target inflammation to protect kidney function and prevent the progression to end-stage renal disease.

### 4.2. Cardiac Involvement and Inflammation in Fabry Disease

In patients affected by FD, cardiovascular complications are the main cause of both reduced quality of life and premature death. Left ventricular hypertrophy (LVH), often resembling hypertrophic cardiomyopathy, is a hallmark of FD cardiac involvement, although glycolipid storage occurs in all cardiac cell types. In the early stages of the disease, the primary cause of cardiac damage is the accumulation of Gb3. Additional processes, such as disrupted energy metabolism, chronic inflammation, and programmed cell death further contribute to cardiac damage and disease progression. Cardiac manifestations in FD, in addition to LVH, often include myocardial fibrosis, heart failure, and arrhythmias [133]. Additionally, cardiac inflammation may contribute to arrhythmias and thromboembolic events, further increasing stroke risk [25]. These complications may be mild or asymptomatic but tend to worsen with age [134]. Increasing evidence suggests the hypothesis that inflammation may play an important role in the early development and progression of Fabry-related cardiomyopathy [25,81]. For instance, altered peptides derived from oxidative stress or from abnormal protein degradation may act as neoantigens, activating the immune system and the UPR can trigger inflammation [25,81]. The buildup of Gb3 and lyso-Gb3 has also been shown to promote a chronic inflammatory state, either by directly acting as a DAMP or by promoting the release of inflammatory mediators [135]. Furthermore, activation of TLR4 may enhance the TGF-*β* response, leading to ECM remodeling and myocardial fibrosis [136]. A large study involving 182 patients of different gender and age, with classical or late-onset FD mutations, helped delineate distinct phases of cardiac involvement [137]. The earliest phase, beginning in childhood, presents myocardial storage without signs of inflammation or LVH. In the next stage, changes in cardiac imaging and biomarkers may appear before structural abnormalities become evident, especially in female patients [137]. Without timely intervention, the disease progresses to advanced stages marked by significant LVH, fibrosis, and myocardial ischemia. A decrease in T1 values, reflecting glycolipid accumulation in the myocardium, and an increase in T2 values, indicating myocardial edema and inflammation, can be detected prior to the onset of LVH [138,139]. The N-terminal pro b-type natriuretic peptide (NT-proBNP) levels typically increase further and symptoms become more apparent [133]. Another early sign of cardiac involvement is impaired myocardial perfusion, reflecting microvascular dysfunction driven by both storage and inflammation [133,140]. Histological studies provide further insight into cardiac pathology. Endomyocardial biopsies from FD patients reveal hypertrophic and disorganized cardiomyocytes, apoptotic cells, and glycosphingolipid deposits. Markers of oxidative stress, including inducible nitric oxide synthase and nitrotyrosine, are often elevated [141]. Additionally, serum levels of pro-inflammatory cytokines, such as IL-6, IL-1β, TNF-α, MCP-1, ICAM-1, and soluble vascular cell adhesion molecule 1 (sVCAM), are significantly higher in FD patients, reinforcing the role of inflammation in disease progression [28]. Further evidence from autopsy showed scattered apoptotic myocytes along with mild infiltration of T-lymphocytes [142]. Notably, the presence of inflammatory macrophages in biopsy samples suggests that these immune cells may be central contributors to the ongoing damage in the hearts of patients with FD [85]. These findings suggest that inflammation may play a crucial role in the progression of Fabry cardiomyopathy. It is still not fully understood whether myocardial inflammation is the primary driver of fibrosis in FD. Moreover, it remains uncertain whether secondary pathways activated by Gb3 accumulation, such as systemic and tissue-level inflammation, can eventually progress independently of ongoing storage. If so, these mechanisms might continue to cause damage even when Gb3 storage is controlled by specific therapies [133]. In conclusion, while Gb3 accumulation can be considered the first step of cardiac involvement in FD, increasing evidence suggests that inflammation plays a key and possibly independent role in driving the disease progression. This highlights the importance of developing treatments that not only reduce storage but also target inflammatory pathways.

### 4.3. Central and Peripheral Nervous System in Fabry Disease: The Role of Inflammation

Neuroinflammation is a common feature in many LSDs and is increasingly recognized as a key factor contributing to neuronal damage and neurodegeneration [143]. In the CNS, glial cells, particularly microglia and astrocytes, play a central role in initiating and regulating inflammatory responses. Depending on the context, this inflammation can be protective or harmful. For instance, a short-term immune response with transient inflammation following CNS injury can be neuroprotective and support repair. However, when inflammation becomes chronic, as often seen in LSDs, it can create a toxic environment that accelerates neuronal dysfunction and death through the release of cytokines, chemokines, and other pro-apoptotic signals [143,144]. Approximately two thirds of patients affected by LSDs exhibit neurological manifestations, highlighting the CNS as a primary target in many of these disorders [145]. In FD, inflammation appears to be one of the driving forces behind the neurological manifestations, although the exact mechanisms remain complex and multifactorial. While direct Gb3 or lyso-Gb3 accumulation in central neurons is not yet fully demonstrated, elevated levels of these metabolites have been found in the plasma and tissues of both FD patients and animal models [146]. Neurological symptoms in FD involve both the CNS and peripheral nervous system (PNS) and arise through various interconnected molecular pathways, with inflammation playing a pivotal role. These symptoms can include acroparesthesia, gastrointestinal dysmotility, and significant cerebrovascular events such as transient ischemic attacks, lacunar infarcts, and white matter hyperintensities, all frequently linked to small vessel disease. The cerebral vasculopathy observed in FD affects both large and small vessels, and ischemic stroke represents one of the most concerning neurological complications. Inflammatory mechanisms contribute to this vasculopathy by promoting endothelial dysfunction, altering the structure and function of vessel walls, and affecting blood components [147,148]. Gb3 and lyso-Gb3 deposition at the vascular level leads to a series of events including smooth muscle cell proliferation, fibrotic remodeling, infiltration of inflammatory cells and activation of the inflammation cascade in the vessel wall [149]. Microglia are the primary neuroinflammatory cells in the CNS, which constantly monitor stress or damage signals [150]. Microglia detect DAMPs released by injured or dying neurons via TLRs and other PRRs [144,151]. In response, they release inflammatory molecules such as cytokines and chemokines, which lead to the recruitment of immune cells at affected sites. Depending on the context, this inflammatory cascade may be protective or harmful, potentially altering cell function or even leading to cell death [152]. Neuroimaging studies in patients have occasionally shown white matter lesions and cerebral microinfarcts, which may be linked not only to vascular abnormalities but also to local inflammatory processes [153]. Notably, cerebral small vessel disease (SVD), a complication of the CNS, is characterized by white matter hyperintensities, cerebral microbleeds, and cerebral infarcts. SVD is frequently observed in brain magnetic resonance imaging (MRI) scans of FD patients, often preceding clinical stroke symptoms by many years. One study, conducted on a cohort of 21 FD patients, reported that the prevalence of SVD was three times higher than the prevalence of stroke symptoms, with changes observable as early as the fifth decade of life [153]. The PNS is also significantly affected in FD. The gradual accumulation of Gb3 in dorsal root ganglia (DRG) neurons and Schwann cells contributes to small fiber neuropathy, one of the earliest and most common symptoms of FD [154]. Patients frequently report chronic pain, burning sensations, and altered thermal and pain perception. Additional features may include hypohidrosis, gastrointestinal disturbances such as abdominal pain and impaired intestinal motility, and cardiovascular abnormalities, particularly arrhythmias [155,156]. Importantly, inflammation may also contribute to PNS dysfunction. Studies have shown that FD patients exhibit a systemic pro-inflammatory profile, which may have an immune-mediated component that exacerbates neuropathy and pain [29,82]. Elevated levels of pro-inflammatory cytokines, such as TNF-α and IL-1β, in the serum and tissues of FD patients are thought to sensitize nociceptive neurons, contributing to neuropathic pain [157]. While normal pain transmission relies on classical neurotransmitter-based neuron-to-neuron communication, pathological pain often involves immune cells, glial cells (astrocytes and microglia), and inflammatory mediators. These components disrupt normal neuronal signaling, leading to chronic pain conditions observed in many FD patients [158,159,160,161]. Collectively, these findings support the concept that inflammation, through both systemic and localized mechanisms, significantly contributes to the development and progression of neurological manifestations in FD, affecting both the central and PNS. Considering inflammation not only as a secondary consequence but also as an active driver of neuropathology may be helpful for developing new therapeutic strategies. Targeting specific inflammatory pathways, whether through modulation of glial cell activity, cytokine signaling, or vascular inflammation, may offer promising approaches to alleviate neurological symptoms and improve patient outcomes.

## 5. Conclusions

Recent advances have considerably increased our understanding of FD revealing that inflammation plays a central role in disease progression. Traditionally classified as LSD, primarily due to glycosphingolipid accumulation, FD is now increasingly recognized as a condition in which chronic inflammation significantly contributes to organ dysfunction and clinical outcomes. The persistent accumulation of Gb3 and its derivative lyso-Gb3 triggers a cascade of cellular events, including lysosomal dysfunction, mitochondrial impairment, ER stress, and activation of the innate immune system. Together, these processes promote a sustained pro-inflammatory environment, which exacerbates tissue injury and drives the development of fibrosis across multiple organ systems. Several key inflammatory pathways have been identified, notably those involving TLRs receptors, oxidative stress mechanisms, and the UPR. Chronic activation of NF-κB signaling, formation of inflammasomes, and dysregulation of cytokine networks have been consistently observed both in patient tissues and in circulating immune cells. These findings underline that inflammation is not a secondary effect but rather a fundamental response involved in organ damage in FD. However, the clinical heterogeneity observed in FD suggests that the inflammatory response may persist independently of substrate storage, especially in advanced disease stages, highlighting the complexity of immune activation. The identification and validation of new biomarkers to monitor inflammatory processes could be important for the development of personalized and effective treatment strategies. Furthermore, recent advances in the field of immunomodulation have highlighted the potential of targeting not only classical inflammatory pathways but also epigenetic regulators to mitigate chronic inflammation in FD and related LSDs. Beyond canonical approaches, such as the inhibition of TLR4 signaling, new small molecules acting at the epigenetic level are being actively investigated. Among these, Apabetalone (RVX-208), a selective bromodomain and extraterminal domain (BET) inhibitor, has emerged as a promising candidate. Apabetalone modulates the expression of pro-inflammatory and pro-fibrotic genes by interfering with BET protein-mediated chromatin remodeling and transcriptional regulation, reducing systemic inflammation and vascular dysfunction [162,163]. Preclinical studies and early-phase clinical trials have shown that RVX-208 decreases levels of inflammatory cytokines, attenuates vascular calcification, and improves endothelial function in patients with high cardiovascular risk profiles [162]. These findings suggest that epigenetic modulators could be helpful as adjunctive therapies to ERT, potentially targeting the residual inflammation that persists despite glycosphingolipid reduction and improving clinical outcomes. As for other LSDs, such as Gaucher disease, where immunomodulatory approaches have shown encouraging results, also for FD, integrating anti-inflammatory and epigenetic modulators, as adjunctive therapies, could be helpful to further improve patient outcomes targeting both substrate accumulation and chronic inflammation [164]. In conclusion, FD should now be viewed not only as LSD but also as a chronic inflammatory condition with systemic effects. Integration of anti-inflammatory strategies with conventional therapies may further enhance patient outcomes in FD. A deeper understanding of the inflammatory mechanisms in FD is essential not only for advancing our knowledge of disease pathogenesis but also for developing novel mechanism-based therapies.

## Data Availability

Not applicable.
